# Bio‐Based Solar Energy Harvesting for Onsite Mobile Optical Temperature Sensing in Smart Cities

**DOI:** 10.1002/advs.202305294

**Published:** 2023-09-15

**Authors:** Sandra F. H. Correia, Ana R. N. Bastos, Margarida Martins, Inês P. E. Macário, Telma Veloso, Joana L. Pereira, João A. P. Coutinho, Sónia P. M. Ventura, Paulo S. André, Rute A. S. Ferreira

*Adv. Sci*. **2022**, *9*, 2104801

DOI: 10.1002/advs.202104801

In the published article, the building icon on Figure 1b and in the table of content figure was first published by Meinardi et al. in *Nature Reviews Materials* and thus this publisher holds its copyright. The correct figure caption and the additional reference can be found below.

Figure 1. a) Scheme of ZEB implementation fundamentals. b) Schematic representation of the building integrated luminescent solar concentrators (LSCs) as double‐glazed windows attached to Si photovoltaic (PV) cells with intrinsic ability to monitor temperature. Building icon: Adapted with permission.^[52]^ Copyright 2017, Springer Nature. LSCs consist of planar waveguides doped or coated with emissive materials that absorb sunlight and re‐emit it at distinct wavelengths. The emitted light is guided toward PV cells coupled to the edges and converted into electricity. The red arrows inside the LSC indicate the total internal reflection of the emitted light. c) Scheme of data transmission to a user‐friendly app/website.

[52] F. Meinardi, F. Bruni, S. Brovelli, *Nat. Rev. Mater*. **2017**, *2*, 17072.

